# Prognostic Value of Performance Status, Albumin, and CRP in Last-Line Chemotherapy for Pancreatic vs. Other Gastrointestinal Cancers—Simple Tools Matter

**DOI:** 10.3390/curroncol31090404

**Published:** 2024-09-14

**Authors:** Arne Westgaard, Aleksandra Pirnat, Marianne Jensen Hjermstad, Nina Aass, Stein Kaasa, Olav Faisal Dajani

**Affiliations:** 1 Department of Oncology, Oslo University Hospital, 0424 Oslo, Norwayn.k.aass@medisin.uio.no (N.A.); uxolaj@ous-hf.no (O.F.D.); 2 European Palliative Care Research Centre (PRC), Department of Oncology, Oslo University Hospital and Institute of Clinical Medicine, University of Oslo, 0318 Oslo, Norway

**Keywords:** pancreatic adenocarcinoma, gastrointestinal cancer, end-of-life chemotherapy, palliative care

## Abstract

Patients with advanced gastrointestinal cancers often receive chemotherapy near the end of life (EoL), raising concerns about overtreatment. The PALLiON trial, a cluster-randomized trial, assessed the impact of a complex intervention on frequency of EoL treatment; the intervention involved palliative care referrals and the use of PROMs. The present secondary analysis evaluated the prognostic value of baseline performance status (PS), albumin (alb), C-reactive protein (CRP), and body mass index (BMI) for overall survival, comparing pancreatic (PAN, n = 189) vs. other gastrointestinal cancer patients (GI, n = 286). Baseline PS, alb, CRP, mGPS (modified Glasgow prognostic score), and BMI were analyzed using Cox regression. Adjusted for age, sex, and hospital size, PS ≥ 2 and alb < 35 g/L predicted shorter survival in both PAN and GI cancers, while CRP > 10 predicted shorter survival only in GI cancers. In PAN, PS ≥ 2 predicted a 78.4% higher probability of shorter survival, and mGPS 2 predicted a 68.7% higher probability. In GI, mGPS 2 predicted a 70.8% higher probability, whereas PS was not significant. BMI did not improve predictive models. PS ≥ 2 and low albumin are strong predictors of short survival in PAN, whereas increased CRP and low albumin (mGPS 2) are predictors in GI.

## 1. Introduction

Patients with pancreatic (PAN) and other gastrointestinal (GI) cancers often receive systemic treatment near the end of life (EoL), raising concerns about overtreatment. Prognosis varies significantly due to factors like tumour biology, patient comorbidities, and treatment responses. In patients with advanced cancer, treatment decisions must balance life extension benefits with therapy risks and burdens to the patients and the family. A nuanced approach is essential to prioritize the patient’s quality of life, avoid unnecessary interventions, and minimize hospital visits whenever possible [1].

Norway’s public healthcare system provides universal health care for all cancer patients. Despite Norway being frequently ranked among the best public health systems globally [2,3], there is a notable variation at the national level in the timing of cessation of EoL chemotherapy for PAN across Norwegian university hospitals [4]. This underscores the need for improved prognostic tools to guide therapeutic decision making, balance patient information about effects and side effects, improve shared decision making, and optimize patient management towards the end of life.

Research has identified various physiological and biochemical markers as potential prognostic tools in cancer patients. Performance status (PS), albumin levels, C-reactive protein (CRP), and body mass index (BMI) correlate with survival outcomes across cancer types. Tools combining these variables aim to predict survival times and tailor treatments [5]. Notable among these is the modified Glasgow prognostic score (mGPS), which was established as a prognostic tool across multiple cancers [6]. This tool is based on the CRP and albumin levels at the time of diagnosis of advanced cancer. The original trial evaluated GPS in multiple cancers, including hepatopancreaticobiliary cancers, and has since been examined repeatedly in pancreatic cancer in various settings, with contradictory results [7]. Few studies, however, have evaluated the prognostic role of mGPS specifically at start of last-line chemotherapy for pancreatic cancer. Furthermore, the predictive accuracy of prognostic markers may vary significantly among different gastrointestinal cancers due to biological differences, with pancreatic cancer displaying particularly aggressive features [8]. A single parameter like performance status, albumin, or CRP may be more feasible for clinical decision making if it predicts survival as well as combined tools.

The PALLiON trial was a cluster-randomized controlled trial aiming to examine the effect of a complex intervention with time-based vs. needs-based referrals to palliative care and use of patient-reported outcome measures (PROMs) on the provision of EoL anticancer treatment. Patients at 12 Norwegian hospitals (2017–2022) were included at the start of last-line chemotherapy according to well-established national treatment guidelines, and they were followed until death [9]. The intervention comprised compulsory referral to palliative care at inclusion and regular PROMs evaluated in structured consultations supplemented by information about treatment intent and evaluations. The primary outcome was overall use, start, and cessation of anticancer therapy in the last 3 months before death. Results did not show any significant differences in the probability of receiving EoL therapy between the intervention hospitals and hospitals that delivered standard care.

**Figure 1:**
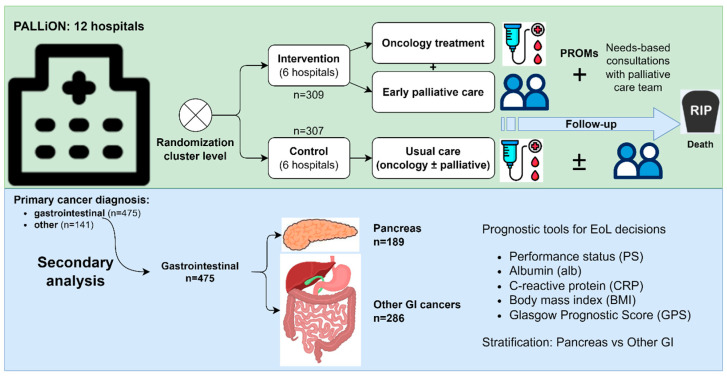
The PALLiON trial assessed time-based vs. needs-based referrals to palliative care and use of PROMs, following patients from start of last-line chemotherapy until death. In the present study, we retrospectively compared PS, CRP, mGPS (modified GPS), and BMI as independent predictors of overall survival in advanced pancreatic cancer and other gastrointestinal cancers.

The present secondary analysis of the PALLiON trial aimed to compare performance status (PS), albumin (alb), CRP, and mGPS, along with body mass index (BMI). The objective was to evaluate the utility of the above-mentioned as independent predictors of overall survival from start of last-line chemotherapy until death for advanced pancreatic cancer (PAN) and other gastrointestinal (GI) cancers (Figure 1).

## 2. Materials and Methods

### 2.1. Study Design

The PALLiON trial followed 616 patients from the start of last-line chemotherapy until death across 12 Norwegian hospitals (2017–2022) [9]. It included adults eligible for last-line systemic treatment for incurable cancers of the upper and lower gastrointestinal (GI) tract, pancreas, liver, biliary duct, gallbladder, breast, bladder, prostate, kidney, or malignant melanoma. Among these, 189 were PAN (30.7%), 286 were other GI, and 141 were other malignancies. The present study was a post hoc analysis comparing PAN vs. other GI cancers, with complete follow-up of the patients until their death. More information on the original sample can be found in Hjermstad et al. [9].

### 2.2. Prognostic Factors

We assessed the utility of baseline PS, alb, CRP, mGPS, and BMI as prognostic factors for overall survival from the start of last-line chemotherapy until death for PAN and GI. Data were collected prospectively and recorded in the PALLiON trial database [10].

All prognostic factors were recorded at study inclusion before starting the last-line chemotherapy. Albumin values (g/L) were categorized as <35 vs. ≥35, and CRP levels (mg/L) were categorized as >10 vs. ≤10, corresponding to common prognostic study thresholds and mGPS values. We defined a priori the cutpoints for albumin and CRP, aligned with the established cutpoints for mGPS, to avoid potential bias by maximizing the difference between “positive” and “negative” samples and thereby increasing the risk of reporting significant results by chance. mGPS was scored as follows: 0 for CRP ≤ 10, 1 for CRP > 10, and 2 for CRP > 10, and albumin < 35 [6].

PS was classified according to the Eastern Cooperative Oncology Group (ECOG) scale [11], ranging from 0 (fully active) to 5 (dead). The Karnofsky PS scores from the PALLiON trial were converted to ECOG scores using validated criteria [12]: ECOG 0 for Karnofsky 90–100, ECOG 1 for Karnofsky 70–80, ECOG 2 for Karnofsky 50–60, and ECOG 3 for Karnofsky 30–40. No patients scored lower than Karnofsky 30.

### 2.3. Statistical Analyses

Baseline characteristics were presented as means with standard deviations (continuous data) and numbers with percentages (categorical data). Age was a continuous variable, while sex (male/female), PS, BMI (<18.5 and ≥18.5) at inclusion, and hospital size were categorical variables. Hospital size, based on the population within the local catchment area, was categorized a priori in the PALLiON trial to minimize the imbalance, and was as follows: small (69,000–136,000 inhabitants), medium (169,000–282,000), and large (300,000–492,000). Prior to secondary analysis, patients were grouped in PAN versus GI to compare the utility of the abovementioned prognostic factors between these different cancers.

Survival time was defined as the number of days from the start of the last chemotherapy line until death. We used the Cox proportional hazards method to model survival time and calculate hazard ratios (HRs). The basic model included adjustments for age, sex, diagnosis (for GI cancers), PS, and hospital size. We explored the contribution of different prognostic factors by adding predictors in separate models. The probability of shorter survival was assessed using the formula P = HR/(1 + HR) [13]. Missing values are detailed in table captions/footnotes. Statistical significance was set at *p* < 0.05 for all tests.

### 2.4. Ethical Approval and Consent

All patients provided written informed consent. The study was approved by the Regional Committee for Medical and Health Research Ethics, South-East Norway (2016/1220-PALLiON), the Data Protection Official at Oslo University Hospital (OUS), and the hospitals’ Institutional Review Boards. It was registered at ClinicalTrials.gov (No. NCT03088202) in March 2017.

## 3. Results

There were no significant differences in baseline characteristics between the groups (PAN, n = 189; GI, n = 286) in terms of age, sex, PS (≥2 vs. 0–1), BMI (<18.5 vs. ≥18.5), albumin (<35 vs. ≥35 g/L), CRP (>10 vs. ≤10), and hospital size (small/medium/large) (Table 1).

**Figure 2:**
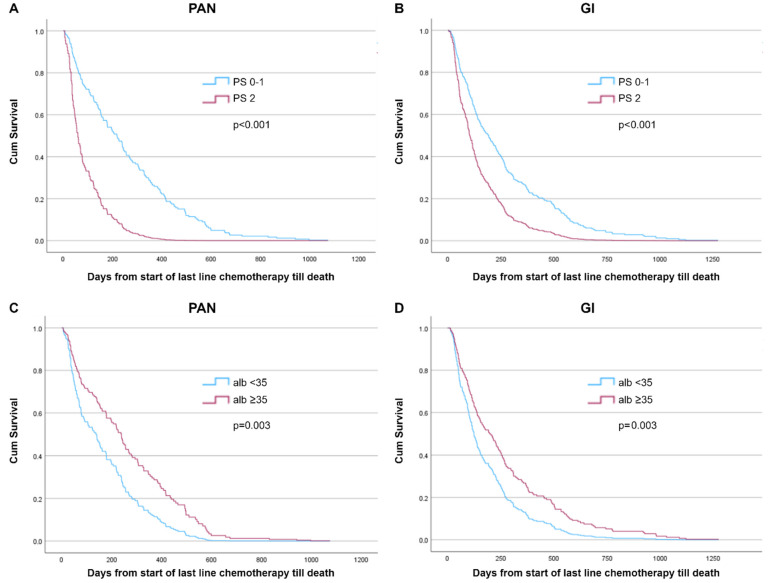
Cox-adjusted survival curves from start of last-line chemotherapy until death by baseline performance status (PS) and albumin (alb, ≥35 g/L vs. <35 g/L) among patients with pancreatic cancer (PAN; n = 189) and other gastrointestinal (GI) cancers (n = 289). (**A**) PAN, PS ≥ 2 vs. 0–1; *p* < 0.001. (**B**) GI, PS ≥ 2 vs. 0–1; *p* < 0.001. (**C**) PAN, alb ≥ 35 g/L vs. <35 g/L; *p* = 0.003. (**D**) GI, alb ≥ 35 g/L vs. <35 g/L; *p* = 0.003. Model adjusted for age, sex, and hospital catchment area, and additionally for PS at baseline (two categories, (**C**)), and albumin (two categories, (**D**)).

**Figure 3:**
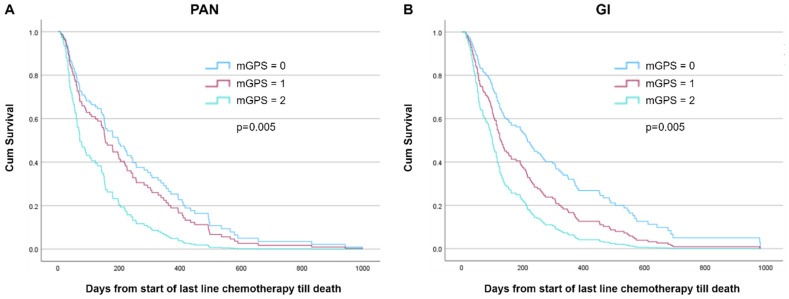
Cox-adjusted survival curves from start of last-line chemotherapy until death by mGPS at baseline among patients with (**A**) pancreatic cancer (PAN; n = 107, *p* = 0.005) and (**B**) other gastrointestinal cancers (GI; n = 149, *p* = 0.005). Model adjusted for age, sex, hospital catchment area, performance status at baseline (2 categories, PS 2–3 vs. PS 0–1), and mGPS (0, CRP ≤ 10 [reference]; 1, CRP > 10; 2, CRP > 10 and alb < 35 g/L).

To evaluate predictors and their association with overall survival from the start of last-line chemotherapy until death, we explored models within PAN and GI groups based on baseline PS, albumin, CRP, and mGPS in a basic model (adjustments for age, sex, hospital size, and cancer diagnosis; Table 2, and Supplementary Table S1). In the PAN group, significant predictors were shown to be PS ≥ 2 (HR 3.38), albumin < 35 g/L (HR 1.74), and mGPS (HR 2.20) (all *p* < 0.05), but not CRP > 10 (*p* = 0.81). As for the GI group, significant predictors included PS ≥ 2 (HR 1.90), albumin < 35 g/L (HR 1.55), CRP > 10 (HR 2.09), and mGPS (HR 2.24) (all *p* < 0.05). Survival curves for PS and albumin and mGPS are shown in Figure 2 and Figure 3, respectively.

We further evaluated PS and mGPS by adding each of the predictors to the basic models by groups and estimated their HR as well as their probability of shorter survival (Table 3, Supplementary Table S2). In the PAN group, PS 2 (vs. 0–1) predicted a 78.4% probability of shorter survival (*p* = 0.002) when added to the basic model and mGPS, while mGPS 2 (vs. 0–1) predicted a 68.7% probability of shorter survival (*p* = 0.005) when added to the basic model and PS. In the GI group, PS 2 (vs. 0–1) was not significantly associated with overall survival (*p* = 0.70) when added to the basic model and mGPS, whereas mGPS 2 (vs. 0–1) predicted a 70.8% probability of shorter survival (*p* = 0.002) when added to the basic model and PS.

Including BMI in the multivariate models did not enhance their predictive strength (Supplementary Tables S1 and S2). BMI was neither associated with overall survival in the PAN group (*p* = 0.74) nor in the GI group (*p* = 0.39) when adjusted for the basic model and PS.

## 4. Discussion

Pancreatic cancer and other gastrointestinal cancers present distinct challenges in clinical management, particularly as patients often receive systemic treatment near the end of life, raising concerns about overtreatment [14,15]. Physicians’ ability to predict survival is often overly optimistic, highlighting the need for prognostic tools such as PS, laboratory findings, or more complex prognostic tools tailored to specific cancer diagnoses and treatment settings [1].

Norway’s public healthcare system provides cancer treatment in accordance with national guidelines for anticancer therapy. In spite of this, there is notable variation at the national level in the timing of cessation of EoL chemotherapy for pancreatic cancer across Norwegian university hospitals, with EoL treatment in the last four weeks ranging from 4% to 22% at Norwegian university hospitals for patients treated in 2022–2023, with similar differences when expanding the period to 2019–2023 [4]. In the PALLiON study [9], the palliative intervention did not significantly influence the frequency of chemotherapy delivered during the last 3 months, 2 months, or 1 month before death for either pancreatic or colorectal cancer patients. This may reflect that relatively few receive EoL anticancer systemic therapy in Norway, although the variation between hospitals does emphasize the need for tools and rules to prevent futile use of chemotherapy for those patients that have a low probability of benefiting from such treatment.

### 4.1. Comparison with Previous Studies

Previous reports on the value of PS, albumin, CRP, mGPS, and BMI in predicting overall survival in pancreatic and other gastrointestinal cancers have mostly included patients at an early stage of anticancer therapy. A strength of the present study is its focus on patients starting the presumptive last-line chemotherapy, for whom avoiding futile treatment is paramount. Furthermore, the prospective data collection in a clinical trial covering hospitals all across Norway, both smaller local hospitals and larger university hospitals, with and without integrated palliative care, represents “real-life” data and their utility as prognostic tools for end-of-life treatment decisions.

The study clearly demonstrates that in this setting, PS ≥ 2 and low albumin are strong predictors of short survival in PAN, whereas increased CRP and low albumin (mGPS 2) are strong predictors of short survival in GI. Our findings underscore the importance of these factors, all available in daily clinical practice, in guiding therapeutic decisions to cease anticancer therapy and transition to a palliative care approach for patients and their families. The present study adds evidence to support that clinicians should be cautious when considering chemotherapy for patients with pancreatic cancer with PS ≥ 2 or for patients with other gastrointestinal cancers with mGPS 2.

Consistent with previous research, our results demonstrate the prognostic significance of PS and albumin levels in both PAN and GI [16]. A PS of 2 or higher was associated with significantly shorter survival in both cancer types, highlighting the detrimental impact of poor functional status on cancer prognosis [11]. Similarly, low albumin levels (<35 g/L) were predictive of shorter survival in both groups. Interestingly, CRP emerged as an independent prognostic factor only in GI cancers, with elevated CRP (>10 mg/L) associated with shorter survival. CRP levels did not independently predict survival in PAN, suggesting differences in the inflammatory milieu between cancer types [17]. Inflammation plays a complex role in PAN and GI cancer progression and response to therapy, requiring further research to clarify the mechanisms behind these differences [8].

The mGPS, encompassing both CRP and albumin levels, proved to be a robust prognostic tool in this study, as demonstrated previously for various other settings [5,6]. Patients with an mGPS of 2, indicative of elevated CRP and low albumin, had significantly shorter survival compared to those with an mGPS of 0 or 1 in both PAN and GI. This highlights the additive prognostic value of combining inflammatory and nutritional markers in predicting outcomes for patients with advanced gastrointestinal malignancies. This may be particularly relevant in non-pancreatic GI cancers for which mGPS 2 was independently associated with overall survival, adjusting for the significance of PS in the final analysis.

In contrast, BMI did not independently predict survival in either PAN or GI cancers when adjusted for other prognostic factors. While BMI is commonly used as a surrogate measure of nutritional status, its utility as an independent prognostic marker in cancer patients is less clear [18]. Our findings suggest that BMI may not offer additional predictive value beyond PS and biochemical markers in this context.

### 4.2. Future Directions

In a recent nationwide population-based cohort study from the US reporting on EoL systemic anticancer therapy from 2015 to 2019, overall survival (OS) was lower for pancreatic cancer patients treated at institutions that delivered the most vs. institutions that delivered less EoL treatment for pancreatic cancer [19]. These differences were not seen for metastatic colorectal cancer.

The benefit of second-line therapy in pancreatic cancer is poorly documented, e.g., two large phase three-trials have provided contradicting evidence on the usefulness of oxaliplatin- and fluorouracil-based combination treatments as a second-line treatment for pancreatic cancer [20,21]. The present study adds to the evidence that the benefit of chemotherapy in pancreatic cancer patients with PS ≥ 2 is very limited (Figure 2A). There is a high risk of overtreatment if patients are not closely monitored with careful assessment of their PS, general condition, and symptoms and how both their disease and treatment affect their quality of life.

The observed differences in prognostic factors between PAN and GI cancers underscore the heterogeneity of gastrointestinal malignancies and the need to differentiate between tumour groups for personalized approaches to patient care. PAN, known for its aggressive behaviour and short life expectancies, appears to be driven by factors such as PS and albumin levels, highlighting the importance of supportive care interventions aimed at optimizing functional performance and nutritional status in this population. In contrast, GI cancers may exhibit a stronger inflammatory component, as evidenced by the prognostic significance of elevated CRP levels. Future studies exploring the underlying molecular pathways driving these differences may provide insights into novel therapeutic targets and strategies for improving outcomes in patients with advanced gastrointestinal malignancies.

### 4.3. Strengths and Limitations

Unlike previous studies focused on earlier treatment stages, our study specifically addresses the prognostic value of these biomarkers in patients starting last-line chemotherapy, a critical juncture for decision making regarding the cessation of anticancer therapy. The results from this study suggest that early identification of patients with PS ≥ 2 or low albumin levels could be instrumental in guiding discussions about transitioning to palliative care earlier in the treatment pathway. Our study does have some limitations that warrant consideration. Firstly, although including only patients eligible for last-line chemotherapy was a strength for the study’s purpose, it may limit the generalisability of our findings to other populations and settings. Additionally, the sample size for certain subgroups, particularly in the GI cancer cohort, may have been insufficient to detect smaller but clinically significant differences in survival.

### 4.4. Clinical Implications

The PALLiON trial was designed to evaluate whether a complex intervention versus usual care could affect start and cessation of anticancer therapy in the last 3 months before death. The main conclusion was that this palliative intervention did not affect the probability of receiving EoL anticancer therapy for all cancer types including pancreatic cancer. The reason may have been that the intervention was not sufficient to have a substantial influence on conventional clinical practice. However, systematic use of PS and albumin, as demonstrated in this secondary analysis, could certainly help clinicians, patients, and caregivers in important EoL treatment discussions. The variation in EoL treatment practices across hospitals highlights the need for standardized protocols that incorporate simple yet effective prognostic tools such as PS and albumin to ensure consistency in decision making.

To address the complex needs of these patients—and to improve the integration of oncological treatment and palliative care—the MyPath project has now been established [22] and is being piloted at several hospitals in Norway and across Europe. Its aim is to equip patients and their families with an efficient and rapid means of reporting symptoms and communicating their needs to healthcare providers, thereby strengthening symptom-targeted treatment and follow-up throughout the cancer care pathway, alongside tumor-directed oncological treatment.

## 5. Conclusions

Our findings highlight the prognostic significance of performance status, albumin, CRP, and mGPS in predicting survival outcomes for patients with advanced pancreatic and gastrointestinal cancers. These findings have important implications for clinical practice, emphasizing the need for comprehensive assessment of these factors to inform treatment decisions and optimize supportive care interventions in palliative settings. Our findings specifically support the integration of PS and albumin assessments into routine clinical practice in pancreatic cancer as simple, accessible tools for guiding EoL decisions. In pancreatic cancer, patients with PS ≥ 2 but who are otherwise considered eligible for last-line chemotherapy should be informed—as a part of shared decision making on whether or not to accept potentially toxic treatment—that life expectancy is limited in this setting. Discussions around the limited benefit of chemotherapy should be prioritized, with a shift toward palliative care to optimize quality of life.

## Figures and Tables

**Table 1:** Baseline clinicopathological characteristics among 475 patients in the PALLiON study before last-line chemotherapy for pancreatic cancer (PAN) or other gastrointestinal cancers (GI).

Characteristics		PAN (N = 189)	GI (N = 286)	*p*
Age	Mean (±SD)	67.7 (8.76)	67.2 (10.24)	0.54
Sex (%)	Male	124 (65.6)	177 (61.9)	0.44
	Female	65 (34.4)	109 (38.1)	
PS (%)	0–1	178 (94.2)	265 (92.7)	
	2	11 (5.8)	19 (6.6)	0.85
	3	0	2 (0.7)	
BMI ^1^ (%)	<18.5	8 (4.2)	14 (4.9)	0.73
	≥18.5	181 (95.8)	271 (94.8)	
Main cancer diagnosis (%)	Pancreas	189 (100.0)	-	NA
	Colorectal	-	131 (45.8)	
	Cholangiocarcinoma	-	57 (19.9)	
	Gastric	-	41 (14.3)	
	Oesophagus	-	35 (12.2)	
	Rectum	-	21 (7.3)	
	Liver	-	1 (0.3)	
mGPS ^2^ (%)	0	29 (15.3)	30 (10.4)	0.47
	1	56 (29.6)	96 (33.6)	
	2	33 (17.5)	46 (16.1)	
Albumin ^3^ (%)	<35 g/L	48 (25.4)	61 (21.3)	0.42
	≥35 g/L	101 (53.4)	158 (55.2)	
CRP ^4^ (%)	≤10 mg/L	31 (16.4)	30 (10.5)	0.08
	>10 mg/L	90 (47.6)	142 (49.6)	
Hospital size (%)	Large	102 (53.9)	149 (52.1)	0.73
	Medium	51 (2.6)	89 (31.1)	
	Small	36 (19.0)	48 (16.8)	

PAN, pancreatic cancers; GI, gastro-intestinal cancers; PS, performance status; BMI, body mass index; CRP, C-reactive protein; NA, not applicable; mGPS score: 0, CRP ≤ 10; 1, CRP > 10; 2, CRP > 10 and alb < 35. Missing data on ^1^ BMI for 1 (0.05%) GI, ^2^ mGPS for 71 (43.4%) PAN and 114 (39.9%) GI, ^3^ albumin for 40 (21.2%) PAN and 67 (23.4%) GI, and ^4^ CRP for 68 (21.2%) PAN and 114 (39.8%) GI.

**Table 2:** Evaluation of prognostic factors in the basic multivariable models *.

	Pancreatic Cancer			Other Gastrointestinal Cancers		
	N	HR (95% CI)	*p*	N	HR (95% CI)	*p*
Performance status						
PS 0–1	178	1.00 Reference		265	1.0 Reference	
PS ≥ 2	11	3.38 (1.75–6.53)	**<0.001**	21	1.90 (1.14–3.15)	**0.01**
Albumin (g/L)						
<35	48	1.74 (1.21–2.50)	**0.003**	61	1.55 (0.93–2.60)	**0.01**
≥35	101	1.0 Reference		158	1.0 Reference	
CRP (mg/L)						
≤10	31	1.00 Reference		30	1.0 Reference	
>10	90	1.48 (0.95–2.90)	0.81	142	2.09 (1.31–3.34)	**0.002**
mGPS						
0	29	1.00 Reference		30	1.0 Reference	
1	56	1.21 (0.73–2.02)	0.46	96	1.57 (0.94–2.62)	0.08
2	33	2.20 (1.26–3.83)	**0.005**	46	2.42 (1.40–4.19)	**0.002**

mGPS score: 0, CRP ≤ 10; 1, CRP > 10; 2, CRP > 10 and alb < 35. Missing data on mGPS for 71 PAN (37.6%) and 114 (39.9%) GI. * Basic model includes adjustments for: age, sex, hospital size, and performance status (PS) at baseline, and also includes adjustment for GI cancer diagnosis. Bold indicates statistical significance.

**Table 3:** Final multivariable models comparing PS and mGPS as independent predictors of overall survival in pancreatic cancer and other gastrointestinal (GI) cancers.

Model	Parameter	p	HR	Probability
	Estimate		(95% CI)	
Pancreatic cancer (N = 189)				
Basic model * + mGPS				
PS ≥ 2	1.29	0.002	3.63 (1.60–8.24)	**78.4%**
mGPS 2	0.79	0.005	2.20 (1.26–3.83)	**68.7%**
Other GI cancers (n = 286)				
Basic model * + mGPS				
PS ≥ 2	0.12	0.70	1.12 (0.62–2.03)	**52.8%**
mGPS 2	0.88	0.002	2.42 (1.40–4.19)	**70.8%**

* Basic model includes adjustments for age, sex, hospital size, and performance status (PS) at baseline, and (for GI) also for cancer diagnosis. PS, performance status; mGPS, modified Glasgow prognostic score (0, CRP ≤ 10 mg/L; 1, CRP > 10 mg/L; 2, CRP > 10 mg/L and albumin < 35 g/L). Bold indicates statistical significance.

## Data Availability

The datasets presented in this article are not readily available due to privacy reasons. Requests to access the datasets should be directed to the corresponding author.

## Supplementary Materials

The following supporting information can be downloaded at: https://www.mdpi.com/article/10.3390/curroncol31090404/s1, Table S1: Basic models used to evaluate the association between clinicopathological factors and survival from initiation of last-line chemotherapy in PAN vs. GI; Table S2: Final multivariable models.
