# Supplementary material for: Prognostic Value of Performance Status, Albumin, and CRP in Last-Line Chemotherapy for Pancreatic vs. Other Gastrointestinal Cancers—Simple Tools Matter
Source: Curr Oncol. 2024 Sep 14;31(9):5462–71. doi: 10.3390/curroncol31090404 (PMC11431321; doi:10.3390/curroncol31090404)
Supplement: Supplementary file 1 [file curroncol-31-00404-s001.zip › curroncol-3181639-supplementary.pdf]

Supplementary Tables S1 and S2

Supplementary Table S1. Basic models to evaluate the association between clinicopathological factors and survival, from initiation of the last line chemotherapy in PAN vs GI

	**Pancreas cancer (PAN)**			**Other gastrointestinal cancers (GI)**		
	Multivariate Model 1			Multivariate Model 2[1]		
	N	HR (95% CI)	P	N	HR (95% CI)	P
Age[2]						
	189	1.01 (0.99-1.03)	.35	286	1.00 (0.99-1.01)	.57
Sex						
Male	124	1.00 Reference		177	1.0 Reference	
Female	65	0.78 (0.58-1.09)	.16	109	1.22 (0.93-1.60)	.15
BMI[3]						
<18.5	8	1.94 (0.94-4.03)		14	0.78 (0.44-1.38)	
≥18.5	181	1.0 Reference	.74	271	1.0 Reference	.39
Performance status						
PS 0-1	178	1.00 Reference		265	1.0 Reference	
PS ≥2	11	3.38 (1.75-6.53)	<.001	21	1.90 (1.14-3.15)	.01
Hospital size[4]						
Large	102	1.00 Reference		149	1.0 Reference	
Medium	51	0.68 (0.47-0.97)	.03	89	0.94 (0.70-1.25)	.67
Small	36	0.83 (0.55-1.25)	.37	48	0.89 (0.63-1.27)	.53
Albumin (g/L)						
<35	48	1.74 (1.21-2.50)	.003	61	1.55 (0.93-2.60)	.01
≥35	101	1.00 Reference		158	1.0 Reference	
mGPS[5]						
0	29	1.00 Reference		30	1.0 Reference	
1	56	1.21 (0.73-2.02)	.46	96	1.57 (0.94-2.62)	.08
2	33	2.20 (1.26-3.83)	.005	46	2.42 (1.40-4.19)	.002
CRP (mg/L)						
≤10	31	1.00 Reference		30	1.0 Reference	
>10	90	1.48 (0.95-2.90)	.81	142	2.09 (1.31-3.34)	.002

PS, performance status; BMI, body mass index; CRP, C-reactive protein; mGPS, modified Glasgow Prognostic Score; CI, confidence interval. [1]Multivariate Model 2 included additional adjustments for cancer diagnosis; [2]Age used as a continuous variable; [3] Missing data on 1 patient; [4] Size refers to the hospital catchment area; [5]mGPS score: 0, CRP ≤10; 1, CRP >10; 2, CRP >10 and alb <35; missing data on mGPS for 71 PAN (37.6%) and 114 (39.9%) GI.

Supplementary Table S2. Final multivariable models

Model	Parameter estimate	P	HR (95% CI)	Probability
**Pancreas cancer (N=189)**				
Basic model*				
PS ≥2	1.22	<.001	3.39 (1.75-6.23)	77.2 %
Basic + BMI				
PS ≥2	1.23	<.001	3.42 (1.77-6.59)	77.4 %
BMI ≥18.5	0.66	.74	1.94 (0.94-4.03)	65.9 %
Basic + Albumin				
PS ≥2	1.21	<.001	3.35 (1.66-6.75)	77 %
Albumin <35 g/L	0.55	0.013	1.74 (1.21-2.59)	63.5 %
Basic + CRP				
PS ≥2	1.31	.002	3.70 (1.64-8.33)	78.7 %
CRP >10 mg/L	0.39	.08	1.48 (0.95-2.29)	59.7 %
Basic + mGPS				
PS ≥2	1.29	.002	3.63 (1.60-8.24)	**78.4 %**
mGPS 2	0.79	.005	2.20 (1.26-3.83)	**68.7 %**
**Other GI cancers (n=286)**				
Basic model**				
PS ≥2	0.64	.014	1.90 (1.14-3.15)	65.5 %
Basic + BMI				
PS ≥2	0.64	.014	1.90 (1.14-3.16)	65.5 %
BMI ≥18	-0.25	.39	0.78 (0.44-1.38)	43.8 %
Basic + Albumin				
PS ≥2	0.44	.13	1.55 (0.88-2.71)	60.8 %
Albumin <35 g/L	0.44	.010	1.55 (1.11-2.16)	60.8 %
Basic + CRP				
PS ≥2	0.18	.54	1.20 (1.64-8.33)	54.5 %
CRP >10 mg/L	0.74	.002	2.09 (1.31-3.34)	67.6 %
Basic + mGPS				
PS ≥2	0.12	.70	1.12 (0.62-2.03)	52.8 %
mGPS 2	0.88	.002	2.42 (1.40-4.19)	**70.8 %**

*Basic model includes adjustments: age, sex, hospital size, and performance status (PS) at baseline. **Basic model for GI includes additionally adjustment for cancer diagnosis. BMI, body mass index; mGPS, modified Glasgow Prognostic Score; CI, confidence interval.
